# Effect of University Social Capital on Working Students’ Dropout Intentions: Insights from Estonia

**DOI:** 10.3390/ejihpe14080160

**Published:** 2024-08-19

**Authors:** Mohammad Abu Sayed Toyon

**Affiliations:** 1 School of Business, European College of Polytechnics, 41531 Jõhvi, Estonia; toyon@ecp.institute; 2 Centre of Management, Estonian Business School, 10114 Tallinn, Estonia

**Keywords:** dropout, higher education, retention, social capital, trust, working student

## Abstract

This study investigates the role of social capital within the university context in retaining working students. It specifically examines the effects of university social capital factors—such as teacher–student relationships, peer networks, and support services—on the dropout intentions of working students, emphasizing the mediating role of employability trust. Using a sample of 1902 working students from the Eurostudent VII survey, this study employed factor analysis techniques and structural equation modeling to derive its findings. The results indicated that university social capital significantly reduces dropout intentions among working students. Strong teacher–student relationships, satisfaction with support services, robust peer networks, and high employability trust positively influence this social capital. There is a statistically significant negative association between teacher–student relationships, peer networks, employability trust, and dropout intentions. Furthermore, the findings reveal that without enhancing students’ employability trust, the effectiveness of support services might be limited. These findings not only contribute to the discourse on student retention and the development of university social capital but also provide practical insights for higher education strategies aimed at supporting working students.

## 1. Introduction

Estonia’s higher education sector faces significant challenges related to dropout rates and graduation timelines, affecting both the labor market and universities’ financial sustainability [1,2]. Recent data reveal an 18.2% increase in university dropouts from 2020 to 2021, followed by a slight decrease in 2022, though numbers remain higher than in 2020, indicating persistent retention issues [3]. Bachelor’s programs saw an 18.7% rise in dropouts from 2020 to 2021, decreasing slightly by 2022 but still 7.4% above 2020 levels. Master’s programs experienced an 18.9% increase in dropouts from 2020 to 2021, with a subsequent decrease in 2022, yet still 2.8% higher than in 2020. Professional higher schools also faced a 4.1% rise in dropouts over two years, highlighting a distinct area of concern [3].

A significant aspect of this issue is the integration of work and study commitments among students [4,5]. Over half of the student population is regularly employed during their education, a figure notably higher than the OECD average [1,6,7]. The number of employed students fluctuated, rising from 22,392 in 2017 to 22,923 in 2018, dropping in 2020, and rebounding to 21,998 in 2021. The employment figures increased from 40,835 in 2020 to 42,614 in 2021, implying that the job market is accommodating student workers or that students are prioritizing jobs over education [8,9,10]. Many students work out of necessity due to financial constraints and high living costs, which, while providing practical experience, often serve as a survival strategy rather than a choice [11,12,13]. Research indicates that working during studies is linked to lower student retention and higher dropout risks, suggesting that working students require targeted support [5,14,15,16]. This trend underscores the necessity for higher education institutions to address the needs of working students, ensuring that their academic and employment responsibilities are balanced effectively.

However, universities face resource constraints and a shift towards revenue-focused models, which jeopardizes investments in building human, social, and cultural capital crucial for student support and success [17]. In this context, social capital [18] becomes especially important, as these students rely heavily on institutional support to balance their academic and work commitments. The prioritization of immediate financial goals over long-term educational objectives, driven by reduced public funding and rising operating costs [18,19,20], often sidelines investment in crucial components of social capital. These components include mentorship programs, access to specialists, student support services, and activities fostering interpersonal relationships among faculty, peers, and staff—all essential for student success and retention. For working students, who already juggle significant responsibilities, the erosion of these support systems can profoundly affect their ability to stay enrolled and succeed academically [14]. In this context, the relationship between social capital within the university and the academic success of working students becomes problematic and is worth investigating.

Indeed, the relationship between social capital and student retention is a compelling area of educational research. For example, research indicates that social capital has a significant influence on college graduation rates, levels of debt, and instances of student loan defaults [21]. Strong relationships between faculty and staff, along with institutional knowledge and trust in the university’s credibility in preparing students for future career opportunities, are crucial for creating a positive academic atmosphere and promoting student achievement. It is especially evident in the first year of college, where the quality of interactions between faculty and students greatly influences their experiences in school [22]. Researchers [23] have also examined the value of friendships among students and concluded that first-year university students who are socially connected are more likely to be retained into their second year. Researchers [24] also showed that the social capital fostered through mentoring relationships positively influences student retention by providing support and guidance. These contributions have significantly advanced the state of knowledge in this field, highlighting the importance of social capital in promoting student persistence. However, much of the existing research focuses on traditional students, leaving a gap in understanding the experiences of working students who combine their studies with jobs. There is a need to explore how social capital affects the success of these students, particularly within the university context. Specifically, it is important to understand how and why components such as teacher–student relationships, peer networks, and support services impact students’ academic survival. Additionally, little is known about the role of employability trust in influencing these students’ success. Investigating how this trust interacts with teacher–student relationships, peer networks, and support services is crucial for understanding its effect on the academic experiences and retention of these students. Therefore, the aim of this current study is to provide insights into the role of social capital within the university context in retaining working students by investigating how teacher–student relationships, peer networks, support service satisfaction, and employability trust influence dropout intentions. This study draws from the theory of social capital [18,25,26,27] and incorporates the framework of university social capital from a prior study [28]. The rest of the paper is structured into several parts, including a literature review, methods, results, discussion, and conclusion.

## 2. Literature Review

Prior studies have highlighted the significance of integrating students socially and academically in order to retain them, and have recommended that institutional policies be developed to fully immerse students in both academic and social aspects of university life [29,30]. Several seminal works [31,32] posited that retention hinges on the integration of students into both the academic and social structures of university life. Academic integration, as scholars [20] argued, involves not only students’ performance and grades but also their interactions with faculty and engagement with the academic aspects of college life, while social integration encompasses students’ involvement in campus life, including relationships with peers and participation in extracurricular activities. These models consider pre-entry attributes such as family background, individual skills, prior educational experiences, and personal motivations, which influence students’ initial commitment to the institution and their educational goals. The strength of a student’s commitment to these goals and the institution shapes their likelihood of persisting in college. Positive experiences within the institution reinforce this commitment, while negative experiences can lead to disengagement and eventual dropout. These theories have been instrumental in understanding the gradual process of student departure, where disengagement can be either academic, due to poor performance or lack of integration, or social, due to a lack of connection to the campus community. Complementing these traditional models, the contemporary model of student retention [33,34,35] emphasizes the importance of students’ psychological processes. These models outline how a student’s background characteristics, interactions with the college environment, psychological processes, and outcomes influence their decision to stay in college. They highlight a feedback loop where institutional experiences can alter a student’s initial characteristics and perceptions, affecting their retention.

While these models have been highly influential, they have faced critiques, particularly regarding their applicability to nontraditional students who might experience college differently [36]. Moreover, these models have been critiqued for focusing too narrowly on campus life and not adequately considering important factors like employability, which are crucial to students’ commitment to higher education. Additionally, they do not fully account for the diverse cultural and social capital that students bring to their educational experiences. Graduate capital, built through the interplay of university social capital, encompasses not just academic achievement but also the development of skills, networks, and attributes that enhance employability and career success, but it is not explicitly addressed in these models. Additionally, research has begun to pivot towards several external factors [36,37]. These expanded views do not ignore what the traditional models have posited, but complement them, as central to this expanded understanding of retention are university social capital factors. Recent research suggests that integration alone may not fully predict retention, highlighting the importance of institutional capital as a critical factor influencing their commitment to higher education [38,39,40,41]. This shift in focus has revealed a gap in understanding how university social capital factors, such as teacher–student relationship, peer network, support service satisfaction, and employability trust, affect dropout intentions, especially among working students. By incorporating the university social capital model into the retention discourse, this study aims to offer actionable insights.

By highlighting the significance of social networks and interactions in acquiring resources, Bourdieu’s theory [25] offers a comprehensive understanding of social capital. He posits that social capital consists of actual or potential resources that individuals or groups gain by having stable networks of institutionalized relationships marked by mutual acquaintance and recognition [28]. Coleman’s approach [18] is especially enlightening in this context, as it emphasizes how social capital promotes cooperation, trust, and shared standards in educational settings [28]. Other scholars [25,26,27,42,43] further expand on the discussion by focusing on the networks, norms, and trust that facilitate coordination and cooperation for mutual benefit. These theories collectively underscore how social capital’s structural, relational, and cognitive dimensions extend beyond individual interactions to include broader community and institutional settings, providing a more comprehensive understanding of how social capital operates within different contexts.

University social capital is a multidimensional construct, encompassing teacher–student relationships, support service satisfaction, peer networks, and employability trust [28]. Within the academic domain, the teacher–student relationship (TSR) is seen as a cornerstone of the educational experience and academic integration [44,45,46]. Recently, the literature has reinforced this view, highlighting the role of TSR in fostering academic engagement and motivation [47]. Peer networks play a vital role in fostering the social integration aspect of student retention. Researchers [38] revealed that social integration, which is enhanced through contacts with peers, has a major influence on a student’s academic experience. The importance of peer interactions in fostering a sense of belonging and receiving support is vital for students’ perseverance, particularly during the transition into the university setting [48,49,50]. The role of support services in student retention cannot be underestimated. Support services act as a bridge between the student and the institution, playing a pivotal role in fostering institutional commitment [51,52].

**Figure 1:**
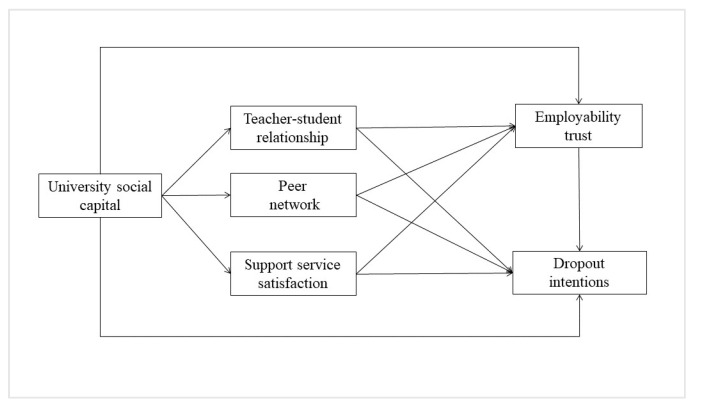
Theoretical model.

Recent studies have shifted attention to how students view their university as a source of capital, particularly in terms of employability [40,53,54]. The emphasis on employability in higher education has led to significant changes in how programs are structured and evaluated [55]. Universities are now tasked with ensuring that their curricula align with industry needs and provide opportunities for students to build the social and cultural capital necessary for workforce success. In an economy where the nature of work is always changing and the abilities needed now might not be the same as those needed tomorrow, this alignment is essential [55]. Moreover, the integration of employability into higher education reflects a broader societal expectation of universities to function not only as educational institutions but also as gateways to career opportunities and economic prosperity. Employability trust has, thus, become highly relevant in the university context. When students place their trust in a higher education institution, they are ultimately relying on the school’s capacity to fulfil its obligations. The students expect that the university will operate to their utmost advantage and conform to expectations that are in line with their educational and vocational ambitions. Making such an investment in trust is not a simple act of belief; it is based on the institution’s proven strengths, its compatibility with student goals, and its ethical behavior. Employability trust in this way extends beyond the academic rigor and reputation of an institution and focuses on the practicality and usefulness of the education obtained in real-world employment situations [28]. It is therefore possible for employability trust to serve as a buffer against dropout intentions, demonstrating that by enhancing students’ belief in their future job prospects, universities can effectively reduce dropout rates and improve overall retention. Given the discussion thus far, it is possible to hypothesize a theoretical model (Figure 1) that illustrates the relationships among various factors influencing working student retention.

The theoretical model shows how university social capital influences students’ dropout intentions through its impact on teacher–student relationships, peer networks, and support service satisfaction. Additionally, these elements directly affect employability trust, which in turn influences dropout intentions. The model emphasizes the relevance of these factors in shaping students’ decisions to remain enrolled. Considering these, the following hypotheses are proposed:

**Hypothesis** **1:** *Teacher–student relationships are positively associated with university social capital*.

**Hypothesis** **2:** *The peer network is positively associated with university social capital*.

**Hypothesis** **3:** *Satisfaction with support services is positively associated with university social capital.*

**Hypothesis** **4:** *Employability trust is positively associated with university social capital.*

**Hypothesis** **5:** *Higher university social capital reduces dropout intentions*.

**Hypothesis** **6:** *Teacher–student relationships positively influence employability trust*.

**Hypothesis** **7:** *Peer networks positively influence employability trust*.

**Hypothesis** **8:** *Satisfaction with support services positively influences employability trust*.

**Hypothesis** **9:** *Employability trust negatively influences dropout intentions*.

**Hypothesis** **10:** *Teacher–student relationships negatively influence dropout intentions*.

**Hypothesis** **11:** *Peer networks negatively influence dropout intentions*.

**Hypothesis** **12:** *Satisfaction with support services negatively influences dropout intentions*.

**Hypothesis** **13:** *Employability trust mediates the relationship between the teacher–student relationship and dropout intentions*.

**Hypothesis** **14:** *Employability trust mediates the relationship between peer networks and dropout intentions*.

**Hypothesis** **15:** *Employability trust mediates the relationship between support service satisfaction and dropout intentions*.

## 3. Materials and Methods

### 3.1. Method

To assess the hypotheses outlined, a quantitative analytical approach was adopted, consisting of the following tasks.

The first task involved conducting factor analysis [56,57], particularly exploratory factor analysis (EFA), to identify and validate the factor structure, showing how items relate to teacher–student relationship (TSR), peer network (PN), support service satisfaction (SS), employability trust (ET), and dropout intentions (DI). In this study, SPSS 23 was utilized for data analysis, applying principal component analysis and varimax rotation. The determination of the number of factors was guided by eigenvalues. The second task used confirmatory factor analysis (CFA) to build on EFA insights by forming and confirming latent constructs, testing hypothesized relationships between observed variables and their corresponding latent constructs, and assessing model fit. This study used AMOS-23 for confirmatory factor analysis (CFA), providing a visual representation and detailed output for evaluating model fit. The fit was assessed using indices such as the comparative fit index (CFI) and Tucker–Lewis index (TLI), with acceptable values being 0.90 or higher. Additionally, the root mean square error of approximation (RMSEA) was used, with values of 0.05 or lower indicating a good fit and values between 0.05 and 0.08 considered reasonable.

The third task involved performing structural equation modeling (SEM) with mediation analysis to uncover the influences of the latent constructs, including TSR, PN, SS, and ET, on dropout intentions. Construct validity, divided into convergent and discriminant validity, was assessed using specific criteria; convergent validity was indicated by a composite reliability (CR) score of 0.7 or higher, while discriminant validity was demonstrated by the average variance extracted (AVE) being higher than the maximum shared squared variance (MSV) and the average shared variance, confirming the test’s distinctiveness and specificity (e.g., [28]).

### 3.2. Data

The data for this study come from the Eurostudent VII survey [58]. This survey was conducted using a comprehensive population survey methodology, and data collection in Estonia took place from February to July 2019 [59]. A total of 1902 working students participated in the survey, offering a vital dataset for analyzing their socioeconomic status in Estonian higher education. In the context of this study, working students refer to individuals enrolled in university who simultaneously engage in employment.

For operationalization in this study, several items from the Eurostudent VII survey were utilized, similar to previous studies (e.g., [28]). For the teacher–student relationship, items included lecturers giving helpful feedback, motivating students to do their best work, being extremely good at explaining things, getting along well with lecturers, and showing interest in what students have to say. For the peer network, the items were knowing many fellow students to discuss subject-related questions and having contact with many students in the study program. Support service satisfaction was measured by satisfaction with support to balance studies and paid job, support to balance studies and family, and support in preparation for future work life. Employability trust was gauged by how well the study program prepares students for the national labor market and the international labor market. Lastly, dropout intentions were assessed by considering whether students were seriously thinking about changing their current main study program and whether they were seriously considering completely abandoning their higher education studies, both measured on a 5-point Likert scale ranging from “Strongly agree” to “Strongly disagree”. Although previous studies have used a similar sample (e.g., [28,60,61]), it is worth describing the sample characteristics used in this study as well.

### 3.3. Characteristics of the Sample

The sample (see Table 1) includes a diverse group of working students, ranging from young adults to those over 30 years old. A significant portion of the sample, approximately 35.9%, consists of mature students aged 30 or older. Additionally, 24.3% are in the 22–25 age bracket, 21.3% are between 25 and 30 years old, and 18.5% are under the age of 21.

The gender distribution reveals that females comprise 76.9% of the respondents, while males make up 23.1%. The predominance of female students might reflect broader trends in gender-based enrollment in higher education in Estonia. The educational levels within the sample are varied. The majority, 57.7% (1098 participants), are enrolled in bachelor’s degree programs (ISCED 6). Master’s degree students represent 36.6% (697 individuals), and a smaller group, 5.6% (107 participants), are pursuing long national degree programs (longer than three years, ISCED 7).

The sample also spans a wide range of academic disciplines. Education accounts for 11.1% (212 individuals), arts and humanities for 16.6% (316 participants), and social sciences, journalism, and information for 13.3% (253 students). The largest group, 19.3% (367 participants), is in business, administration, and law. Natural sciences, mathematics, and statistics are chosen by 6.4% (122 students), ICT by 7.9% (151 students), and engineering, manufacturing, and construction by 5.0% (95 students). The least popular fields are agriculture, forestry, fishery, and veterinary, making up only 0.8% (15 participants). Health and welfare attract 15.4% (293 participants), while 3.9% (75 students) are in service disciplines.

## 4. Results

### 4.1. Results of Exploratory Factor Analysis

The exploratory factor analysis results (see Table 2) from this study reveal several key insights into the constructs being examined. Firstly, the variance explained stands at 70.367%, indicating that the factors effectively capture a significant portion of the underlying patterns in the dataset. Regarding the suitability of the data for factor analysis, the Kaiser–Meyer–Olkin (KMO) measure of sampling adequacy is 0.793, which is well above the recommended threshold of 0.6, suggesting the appropriateness of the sample for this analysis. Additionally, Bartlett’s test of sphericity returns a statistically significant result, confirming the interrelatedness of the variables and the suitability of the data for structure detection.

The factor loadings yield informative results. The teacher–student relationship construct shows high factor loadings for all its items, with a range from 0.733 to 0.782, and Cronbach’s alpha of 0.84, indicating strong internal consistency. The satisfaction with support services construct also demonstrates high factor loadings, ranging from 0.650 to 0.868, coupled with a good Cronbach’s alpha of 0.762. For the peer network construct, the factor loadings are very high, between 0.894 and 0.908, with a Cronbach’s alpha of 0.83, underscoring its reliability. The employability trust construct, with factor loadings between 0.824 and 0.835 and a Cronbach’s alpha of 0.66, confirms a strong association with the items measuring it, although the alpha value is slightly lower than the others. Lastly, the dropout intention construct, indicated by items relating to dropping out or changing programs, has high loadings between 0.834 and 0.852, and a Cronbach’s alpha of 0.65 suggests acceptable reliability.

### 4.2. Results of Confirmatory Factor Analysis and Structured Equation Modeling

**Figure 2:**
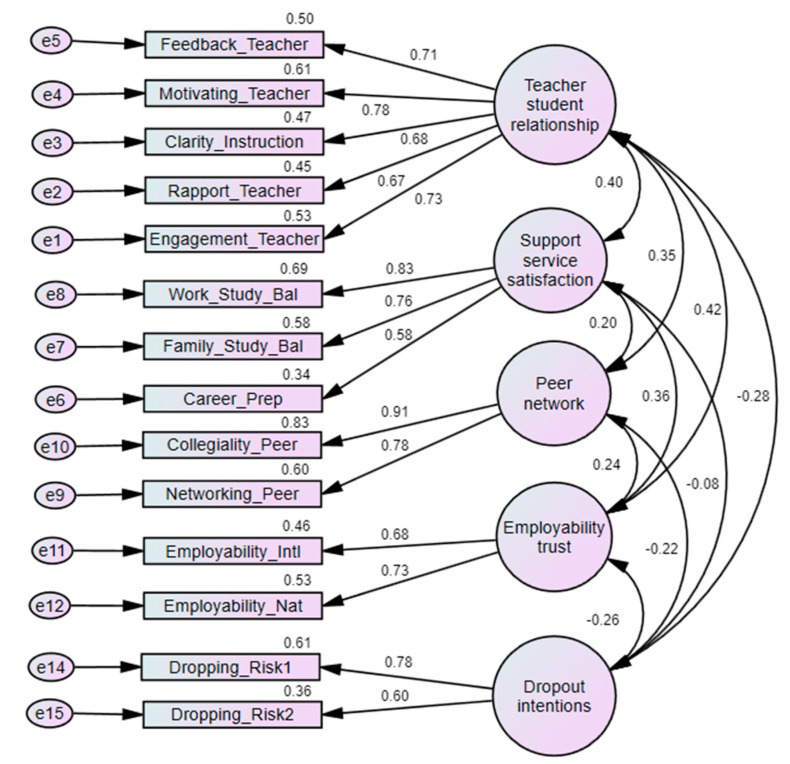
Results of measurement models. Note: Model fitness measures include chi-square = 450.77, *df* = 67, *p =* 0.000, CMIN/DF = 6.728, RMSEA = 0.055, CFI = 0.954, and TLI = 0.938.

After conducting EFA, confirmatory factor analysis was performed to assess the measurement models’ suitability for creating the structural model. Figure 2 demonstrates that the constructs—teacher–student relationship, peer network, support service satisfaction, employability trust, and dropout intentions—exhibit good model fit, with the following values: chi-square = 450.77, *df* = 67, *p* = 0.000, CMIN/DF = 6.728, RMSEA = 0.055, CFI = 0.954, and TLI = 0.938. The discriminant validity (see Table 3) shows the correlations between the constructs. The diagonally bolded values represent the square root of the average variance extracted, while the other values show the intervariable correlations. The bold diagonal values (square root of AVE) are greater than the other values in their respective rows and columns, indicating that discriminant validity is satisfied.

**Figure 3:**
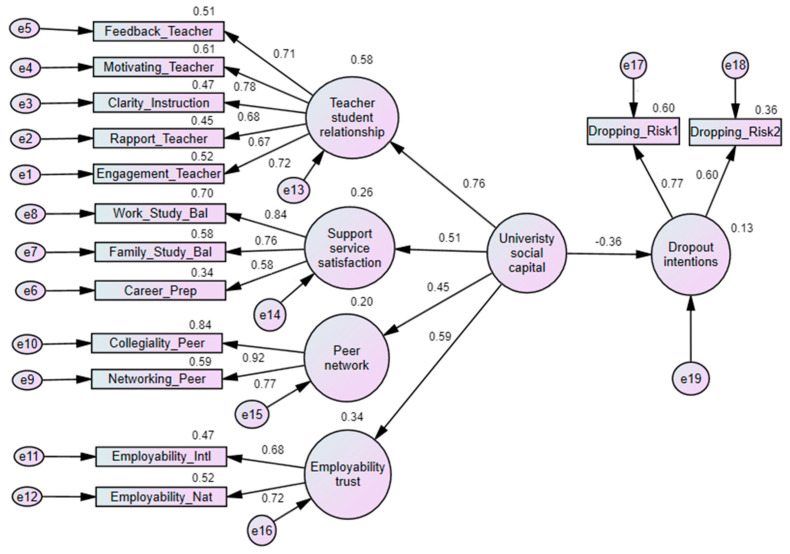
Structural model of university social capital’s impact on dropout intentions. Note: Model fitness measures include chi-square = 487.002, *df* = 72, *p =* 0.000, CMIN/DF = 6.764, RMSEA = 0.055, CFI = 0.951, and TLI = 0.938.

Based on this CFA, two structural models were developed. Figure 3 shows the first structural model. In this structural model, university social capital is associated with the teacher–student relationship, peer network, support service satisfaction, and employability trust, with regression weights of 0.76, 0.45, 0.51, and 0.59, respectively. This model also indicates that university social capital negatively affects the dropout intentions of working students, with a regression weight of −0.36. Specifically, the teacher–student relationship is influenced by the teacher’s motivating skills (regression weight: 0.78), interest and engagement with students (0.72), and feedback (0.71). Support service satisfaction impacts work–study balance service satisfaction (0.84). The peer network construct influences collegiality or connections with students for academic discussion (0.92). Employability trust is significantly influenced by trust in the university’s ability to prepare students for the national labor market (0.72). The model fitness measures indicate a good fit: chi-square = 487.002, *df* = 72, *p* = 0.000, CMIN/DF = 6.764, RMSEA = 0.055, CFI = 0.951, and TLI = 0.938.

**Figure 4:**
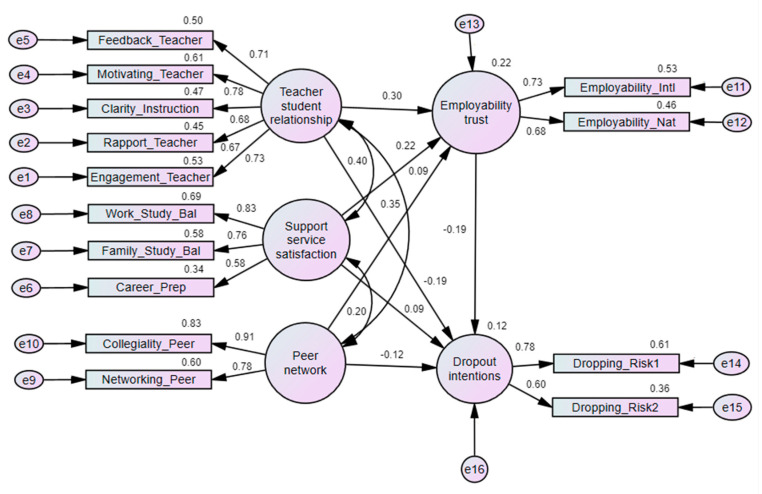
Structural model of the mediation effect of employability trust. Note: Model fitness measures include chi-square = 450.777, *df* = 67, *p =* 0.000, CMIN/DF = 6.728, RMSEA = 0.055, CFI = 0.954, and TLI = 0.938.

To assess the direct effect of the teacher–student relationship, peer network, and support service satisfaction on dropout intention, as well as the role of employability trust in these relationships, another structural model was created, as depicted in Figure 4. In this model, employability trust is influenced by the teacher–student relationship, peer network, and support service satisfaction. Additionally, employability trust also influences dropout intentions. This model shows that employability trust is positively influenced by the teacher–student relationship (regression weight: 0.30), support service satisfaction (0.22), and peer network (0.09). The relevant results of this model are presented in Table 4. The mediation results are also presented in Table 5.

The regression weight (Table 4) indicates several key paths between constructs. Teacher–student relationships (−0.19) negatively predict dropout intentions, indicating that better teacher–student relationships are associated with lower dropout intentions. Similarly, peer network (−0.12) also negatively predicts dropout intentions, meaning that a stronger peer network is associated with lower dropout intentions. On the other hand, support service satisfaction (0.09) positively predicts dropout intentions, which is counterintuitive. Employability trust (−0.19) negatively predicts dropout intentions, showing that higher employability trust is associated with lower dropout intentions. These paths suggest that positive relationships with teachers and peers, as well as confidence in the employability outcomes of education, are crucial for retaining working university students.

Table 5 presents the results of how employability trust (ET) mediates the relationship between teacher–student relationship (TSR), peer network (PN), support service satisfaction (SS), and dropout intentions (DI). For TSR and DI, the total effect of TSR on DI is negative (−0.262 **), indicating that positive teacher–student relationships reduce dropout intentions. The indirect effect (−0.059 *) indicates that ET partially mediates their relationship. The direct effect (−0.203 **) of TSR on DI remains statistically significant, suggesting that while ET explains some of the relationship, TSR independently influences dropout intentions. For SS and DI, the direct effect of SS on DI is positive (0.080 **), unexpectedly suggesting that higher satisfaction with support services is associated with increased dropout intentions. However, when mediated by ET, the indirect effect is negative (−0.036 **), which implies that higher employability trust can mitigate the positive relationship between SS and DI. For PN and DI, PN has a total negative effect on DI (−0.115 **), and this relationship is partially mediated by ET, with an indirect effect (−0.013 **). It implies that a strong peer network can reduce dropout intentions and that this effect is slightly enhanced by employability trust.

## 5. Discussion

The objective of this study was to investigate the role of social capital within the university context in retaining working students. Specifically, it aimed to understand how components such as teacher–student relationship (TSR), peer network (PN), support services (SS), and employability trust (ET) influence students’ academic persistence or dropout intentions (DI). Using data from the Eurostudent VII survey, the study employed factor analysis techniques and structural equation modeling to derive its findings. This study proposed 15 hypotheses, all of which were supported except for hypothesis 12. These findings offer several important insights specific to the Estonian context.

The findings show that university social capital reduces dropout intentions, with a statistically significant negative effect (−0.36) on these intentions. This social capital is positively influenced by strong teacher–student relationships (0.76), satisfaction with support services (0.51), robust peer networks (0.45), and high employability trust (0.59).

Teacher–student relationships are foundational to university social capital. They are built on teachers’ motivation for students (0.78), interest in students (0.72), clarity in instruction (0.68), nurturing faculty–student rapport (0.67), and providing constructive feedback (0.71). Particularly, teachers’ motivation plays a crucial role. Previous studies [62,63,64] have shown that the quality of teaching and classroom management practices affect students’ academic success. In this study, it was found that for working students, the quality of teachers and their teaching practices significantly impact the TSR, which in turn influences dropout intentions. A negative correlation (−0.19) between TSR and DI underscores the importance of strong teacher–student relationships in reducing dropout intentions. While positive TSR alone reduces dropout intentions, employability trust further strengthens this effect by partially mediating the relationship. For working students, who often manage dual responsibilities, supportive and understanding faculty can provide necessary resources, enhancing their commitment to continuing their studies.

Similarly, peer networks are vital for fostering university social capital, significantly affecting student retention. Peer networks facilitate networking (0.91) and collegiality (0.78), providing students with contacts and support within their study programs. A strong peer network directly reduces dropout intentions (−0.12) and enhances employability trust (0.09). Previous studies have highlighted the importance of peer networks for integration into university life, although not all engagement activities are equally effective [65,66]. Working students, constrained by strict time management, seek meaningful connections that support their present and future conditions. For them, the sense of belonging and support derived from peer interactions, such as shared academic resources and study groups, is particularly important. These networks help alleviate the isolation that working students may feel due to limited campus time and divided focus.

Support services also play a crucial role in retaining working students by bridging the gap between students and the institution [62,66]. Students’ satisfaction with support services is reflected in how well they feel supported in balancing work, family, and career preparation. Interestingly, the findings show that higher satisfaction with support services directly correlates (0.09) with increased dropout intentions. However, employability trust mediates this relationship, resulting in a negative indirect effect (−0.036). It suggests that while working students value support services, satisfaction alone does not guarantee retention. Instead, the effectiveness of these services in enhancing employability trust ultimately reduces dropout intentions. Working students often have unique needs, such as flexible scheduling, financial advice, and career counselling tailored to their employment, which standard support services may not fully address.

In this context, employability trust emerges as a critical factor, consistent with findings from a previous study [67]. The mediation role of employability trust indicates that students’ belief in the relevance and effectiveness of their education in securing future employment significantly influences their persistence. From Bourdieu’s perspective [25], this trust acts as symbolic capital for these students. Already in the labor market, they may pursue higher education to advance their careers. Therefore, their belief in education’s relevance and effectiveness in securing better employment opportunities strongly influences their decision to continue their studies. This study highlights that employability trust significantly mediates the relationships between TSR, PN, SS, and DI, emphasizing the need for higher education institutions to align their programs with real-world employment opportunities. It is not just about improving academic quality but also about providing meaningful networking opportunities that directly contribute to employability. This mediation effect of employability trust also reflects the value students place on their educational investment. For many working students, pursuing higher education involves significant financial and personal sacrifices. This study’s findings indicate that when these students trust that their education will lead to better employment opportunities, they are more likely to persist with their studies. In this context, the negative effects of TSR and PN on DI, mediated by employability trust, suggest that strong support from faculty and peers increases students’ confidence in the value of their education, encouraging them to continue their studies.

These findings provide universities with both promising opportunities and significant challenges that demand attention. First, the importance of strong teacher–student relationships and peer networks cannot be overstated. The results clearly show that these relationships play a pivotal role in reducing dropout intentions, particularly among working students who are at risk of feeling isolated. It underscores the necessity of fostering engaging, motivating, and supportive interactions within the academic environment. However, the challenge lies not just in recognizing this importance but also in actively enhancing these relationships, which leads to the next point. Second, improving the quality of the classroom environment is a critical challenge that universities may need to address head-on. While high-quality teaching is fundamental to understanding and improving what happens in the classroom, the current trend towards digitalized learning poses significant obstacles. The shift from face-to-face interactions to digital platforms has the potential to erode the personal connections that are essential for student engagement and retention. It is particularly concerning as these meaningful connections are becoming increasingly virtual, risking a decline in the quality of teacher–student and peer interactions. Therefore, universities may need to explore innovative strategies to maintain and even strengthen these relationships in a digital context.

Third, the managerial implications for universities are profound. Ensuring that the educational environment is conducive to building social capital among students requires more than just maintaining the status quo. University administrators may need to consider investing in targeted teacher training programs that emphasize the importance of interpersonal skills and adaptability to different student needs. Furthermore, they need to recognize the specific challenges faced by working students and tailor the academic environment to support them effectively. This could involve more dedicated support services and an increased focus on creating inclusive classroom dynamics that address the diverse needs of all these students.

Fourth, the relationship between support service satisfaction and dropout intentions introduces a complex challenge. While one might assume that high levels of satisfaction with support services would correlate with lower dropout rates, the findings suggest otherwise. Such a paradox calls for a more comprehensive understanding of what support services are truly effective and how they can be better aligned with the needs of students, particularly those who are integrating academic and professional commitments. Finally, recognizing the role of employability trust as a crucial factor in working students’ retention is essential. Students’ belief that their education will lead to real-world job opportunities needs to be nurtured. This trust is not merely about the quality of the educational programs but also about how well these programs are communicated and perceived by students. If students do not see a clear connection between their studies and their future employability, their commitment to their education may wane, leading to higher dropout rates. It highlights the need for universities to not only design curricula that are closely aligned with job market demands but also to effectively communicate these alignments to students. Thus, while this study highlights promising strategies for enhancing retention through social capital, it also stresses the necessity for a comprehensive support system that addresses the diverse needs of the working student population.

## 6. Conclusions

With ongoing dropout practices from higher education over the past few years, Estonia’s universities continue to grapple with persistent retention challenges. A critical factor contributing to this problem is the high number of students working during their studies due to financial constraints, which is linked to lower retention rates and higher dropout risks. Compounding this issue is the challenge universities face in investing in essential support systems due to limited resources and a shift towards revenue-focused models, which has led to an erosion of the social capital crucial for student success. This study aimed to investigate the role of social capital within the university context in retaining working students. This research provided evidence on how teacher–student relationships impact the retention of working students, in what ways peer networks influence their academic success, how satisfaction with support services affects their dropout intentions, and what role employability trust plays in their retention. By shedding light on these aspects, this study offers insights into enhancing the retention of working students through the strengthening of social capital in universities.

Although this study offers valuable insights, it also has some limitations. For instance, it focuses on dropout intentions rather than actual dropout rates. While understanding dropout intentions helps gauge the effectiveness of existing resources in retaining students, considering actual dropout rates would provide a clearer picture of how well these resources are being utilized within universities. Moreover, this study is correlational and does not account for the longitudinal nature of dropout and retention, which are processes that unfold over time. This study uses cross-sectional data from the Eurostudent VII survey, capturing information at a single point in time. A longitudinal study would be more effective in understanding the gradual impact of related factors on academic completion. Additionally, this study excludes students from distance learning programs, defined here as courses without any physical face-to-face interaction during lectures, which are usually not part of university degree programs. As a result, the focus is specifically on working students enrolled in university degree programs to provide insights that are directly relevant to the university context, where in-person interactions and the integration of work and study play significant roles. However, while the Eurostudent survey may not have specifically aimed to include distance learners, this exclusion represents a future opportunity. Including data from distance learners, even if they fall outside the scope of university degree programs, could have provided a more comprehensive understanding of dropout and retention across various educational formats. Furthermore, the study is centered on working students in Estonia. Comparing this with data from other countries could provide valuable insights into how the situation for working students varies internationally.

## Figures and Tables

**Table 1:** Sample characteristics.

Variables	Frequency	Percent
Gender:		
Female	1463	76.9
Male	439	23.1
Age:		
Up to 21 years	351	18.5
22 to <25 years	463	24.3
25 to <30 years	405	21.3
30 years or over	683	35.9
Parents education:		
Low education background (ISCED 0–2)	118	6.2
Medium education level of parents (ISCED 3–4)	488	25.7
High education level of parents (ISCED 5–8)	1232	64.8
No answer	38	2.0
Don’t know	26	1.4
Qualification:		
Bachelor	1098	57.7
Master	697	36.6
Long national degree	107	5.6
Field of study:		
Education	212	11.1
Arts and humanities	316	16.6
Social sciences, journalism and information	253	13.3
Business, administration and law	367	19.3
Natural sciences, mathematics and statistics	122	6.4
ICTs	151	7.9
Engineering, manufacturing and construction	95	5.0
Agriculture, forestry, fisheries, and veterinary	15	0.8
Health and welfare	293	15.4
Services	75	3.9
No answer	3	0.2
N	1902	100

**Table 2:** Results of exploratory factor analysis.

Item Coding	Items Used for Operationalization	Mean	Standard Deviation	Factor Loading	Cronbach’s Alpha	Composite Reliability	Average Variance Extracted	Maximum Shared Squared
Teacher–student relationship					0.837	0.840	0.510	0.180
Feedback_Teacher	Lecturers give helpful feedback	2.299	1.0502	0.769				
Motivating_Teacher	Lecturers motivate to do best work	2.559	1.0372	0.782				
Clarity_Instruction	Lecturers extremely good at explaining things	2.365	0.8505	0.744				
Rapport_Teacher	Get along well with lecturers	1.823	0.8094	0.733				
Engagement_Teacher	Lecturers interested in what students has to say	2.267	0.9955	.763				
Peer network					0.827	0.830	0.720	0.120
Collegiality_Peer	Know a lot of fellow students to discuss subject-related questions	2.262	1.1398	0.894				
Networking_Peer	Contact with many students in study program	2.391	1.2030	0.908				
Support service satisfaction					0.762	0.780	0.540	0.160
Work_Study_Bal	Satisfaction with support to balance my studies and paid job	3.679	1.5139	0.865				
Family_Study_Bal	Satisfaction with support to balance my studies and family	4.044	1.6595	0.868				
Career_Prep	Satisfaction with support in the preparation for my (future) work life	3.368	1.4783	0.650				
Employability trust					0.656	0.660	0.490	0.180
Employability_Nat	How well the study program prepares for the national labor market	2.485	1.3683	0.835				
Employability_Intl	How well the study program prepares for the international labor market	3.379	1.5663	0.824				
Dropout intentions					0.630	0.650	0.480	0.080
Dropping_Risk1	I am seriously thinking about changing my current main study program	4.492	0.9831	0.834				
Dropping_Risk2	I am seriously thinking of completely abandoning my higher education studies	4.622	0.8731	0.852				

**Table 3:** Measures of discriminate validity.

	TSR	SS	PN	ET	DI
TSR	**0** **.716**				
SS	0.400	**0** **.735**			
PN	0.352	0.201	**0** **.846**		
ET	0.418	0.357	0.235	**0** **.703**	
DI	−0.277	−0.078	−0.218	−0.263	**0** **.695**

Note: TSR = Teacher–student relationship, PN = Peer network, SS = Support service satisfaction, ET = Employability trust, DI = Dropout intentions.

**Table 4:** Regression weights from structural models.

Path			Estimate	Standard Error	Critical Ratio	*p*	Remarks
Teacher student relationship	<---	University social capital	0.758	0.079	11.099	***	Hypothesis 1 supported
Support service satisfaction	<---	University social capital	0.512	0.072	11.214	***	Hypothesis 3 supported
Peer network	<---	University social capital	0.448	0.075	10.163	***	Hypothesis 2 supported
Employability trust	<---	University social capital	0.586	0.103	11.099	***	Hypothesis 4 supported
Dropout intentions	<---	University social capital	−0.361	0.057	−8.767	***	Hypothesis 5 supported
Employability trust	<---	Teacher student relationship	0.3	0.054	8.114	***	Hypothesis 6 supported
Employability trust	<---	Support service satisfaction	0.22	0.043	6.291	***	Hypothesis 8 supported
Employability trust	<---	Peer network	0.085	0.035	2.737	0.006	Hypothesis 7 supported
Dropout intentions	<---	Employability trust	−0.186	0.03	−4.556	***	Hypothesis 9 supported
Dropout intentions	<---	Teacher student relationship	−0.191	0.041	−5.008	***	Hypothesis 10 supported
Dropout intentions	<---	Support service satisfaction	0.091	0.031	2.552	0.011	Hypothesis 12 not supported
Dropout intentions	<---	Peer network	−0.125	0.026	−3.875	***	Hypothesis 11 supported

Note: *** *p* ≤ 0.001.

**Table 5:** Results of mediation analysis.

Path	Total Effects	Direct Effects	Indirect Effects	Remarks
TSR > ET > DI	−0.262 **	−0.203 **	−0.059 *	Hypothesis 13 supported
SS > ET > DI	0.044	0.080 **	−0.036 **	Hypothesis 15 supported
PN > ET > DI	−0.115 **	−0.102 **	−0.013 **	Hypothesis 14 supported

Note: * *p* ≤ 0.05, ** *p* ≤ 0.01; TSR = Teacher–student relationship, PN = Peer network, SS = Support service satisfaction, ET = Employability trust, DI = Dropout intentions.

## Data Availability

Although the data contract does not permit the author to share the data publicly, it is possible to create a separate data contract to gain access to the data through the Centre for Higher Education Research and Science Studies (DZHW).
